# Renal Cell Carcinoma with Direct Extension into the Gonadal Vein, Uterus, Fallopian Tube, and Bilateral Ovaries: A Case Report

**DOI:** 10.15586/jkcvhl.2020.130

**Published:** 2020-08-14

**Authors:** Sarah E. Sweigert, Petar Bajic, Alessa Aragao, Maria Picken, Michael E. Woods

**Affiliations:** 1 Department of Urology, Stritch School of Medicine, Loyola University Chicago, Maywood, IL, USA;; 2 Glickman Urological and Kidney Institute, Cleveland Clinic, Cleveland, OH, USA;; 3 Department of Pathology, Stritch School of Medicine, Loyola University Chicago, Maywood, IL, USA

**Keywords:** gonadal vein, invasive, kidney cancer, ovary, renal cell carcinoma, uterus

## Abstract

Renal cell carcinoma (RCC) with invasion into the renal vein is well described; however, invasion into the gonadal vein is a rare event with less than five cases reported in the literature. RCC occasionally presents with metastasis to the ovaries or the fallopian tubes, although this is also a rare occurrence. We present a case of locally advanced left RCC with direct extension into the ipsilateral gonadal vein with extension into the bilateral ovaries and uterus, which has not been previously described. Computed tomography (CT) in a 72-year-old female with a 35-pound weight loss indicated the presence of a 16-cm left renal mass with caudal tumor extension through the left gonadal vein and regional lymphadenopathy. There was no evidence of distant metastasis, and she underwent an open left radical nephrectomy. Intraoperatively, she was found to have direct extension of the tumor through the left gonadal vein into the uterus, bilateral ovaries, and the left fallopian tube. All visible disease was resected, and retroperitoneal and pelvic lymphadenectomy were performed. The patient had an uneventful hospital course. Pathology revealed clear cell RCC, Fuhrman grade 3. The final pathologic stage was pT4N1M1. The patient was ultimately noted to have pulmonary metastasis and was treated with immunotherapy with no evidence of disease progression.

## Introduction

With 403,262 annual cases around the world, kidney cancer is the sixth most common cancer diagnosis in the United States (1, 2). Renal cell carcinoma (RCC) accounts for over 90% of kidney cancers, and its extension into the renal vein is a common phenomenon. However, extension into the gonadal vein is a rare event with less than 10 case reports available in the literature (3–10). Occasionally, RCC presents with metastasis to the ovaries or the fallopian tubes, although this is also a rare occurrence (11, 12). To our knowledge, there have been no reported cases of a RCC with extension into the gonadal vein with contiguous invasion into the bilateral ovaries, fallopian tube, and uterus. Here, we present such a case with informed consent having been obtained from the patient. The present case report was deemed exempt from the institutional review board due to the de-identified nature of the information presented.

## Case Presentation

**Figure 1:**
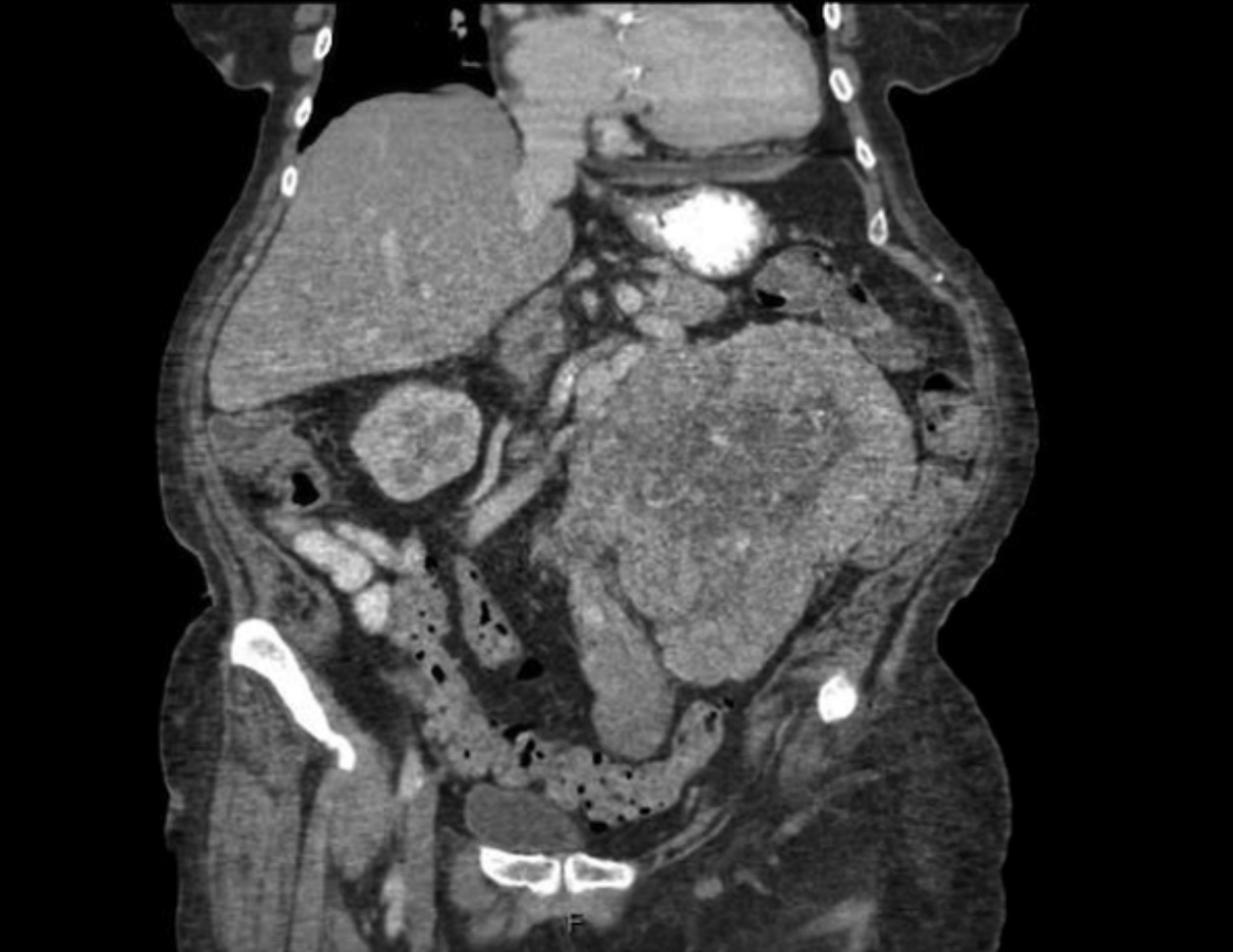
Coronal view of computed tomography of abdomen/pelvis revealing a large left renal mass with direct extension into the left gonadal vein.

A 72-year-old female presented to her primary care physician reporting progressive fatigue, abdominal pain, and a 35-pound unintentional weight loss. She was noted to have a palpable left abdominal mass, and laboratory work was notable for hypercalcemia, thrombocytosis, and elevated alkaline phosphatase. Subsequent computed tomography (CT) imaging revealed a 16-cm left-sided renal mass with caudal tumor extension and bulky regional lymphadenopathy along the left gonadal vein (Figure 1). The patient was then referred to our urology department for further management.

**Figure 2:**
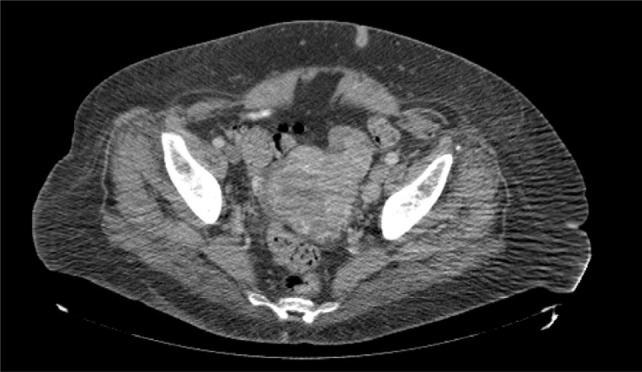
Axial view of computed tomography of the abdomen and the pelvis revealing a heterogeneous appearance of the uterus.

The patient’s medical and surgical history was unremarkable except for insulin independent diabetes mellitus. A left-sided abdominal mass was palpable on physical exam. Further evaluation revealed no evidence of distant metastatic disease on CT imaging of the head, chest x-ray, recent CT chest, or CT abdomen and pelvis. Magnetic resonance imaging of the abdomen, obtained approximately 1 month prior to her surgery, did not reveal any evidence of left renal vein involvement. After extensive counseling and a thorough discussion of the risks involved and the benefits, she opted for surgical extirpation of her locally advanced renal mass and regional lymphadenopathy. She underwent an open left radical nephrectomy, total abdominal hysterectomy with bilateral salpingo-oophorectomy, and retroperitoneal and pelvic lymph node dissection. Intraoperatively, the mass was found to be large and bulky, extending toward the pelvis and invading the local structures. The ascending colon was reflected medially, at which time the mass was noted to be involving part of the mesentery, which required resection. Once the hilum was controlled, the mass was dissected down toward the pelvis and was noted to be involving the uterus and the bilateral ovaries. A supracervical hysterectomy with bilateral salpingo-oophorectomy was performed. The left gonadal vein with tumor thrombus was mobilized and the mass was dissected off the lateral abdominal wall, which allowed the whole specimen to be elevated en bloc until it was completely free. A retroperitoneal and left pelvic lymph node dissection was completed, and all specimens were sent for pathologic examination. Estimated blood loss was 750 mL, and there were no intraoperative or postoperative complications. The patient had an uneventful 5-day hospital course and was discharged to a subacute rehabilitation facility. Upon further review of the abdominal and pelvic CT scan, there was a heterogeneous appearance of the uterus, which had been assumed to be related to fibroids (Figure 2).

Repeat CT chest postoperatively revealed several suspicious bilateral lung nodules, which were not seen preoperatively. Further staging was performed, which showed no other evidence of metastasis. Given the new finding of distant metastasis, the patient elected for combination immunotherapy with four cycles of ipilimumab and nivolumab, which was recently completed. The most recent imaging showed resolution of the right-sided lung nodules, stable left lung nodules, and no new sites of metastatic disease.

## Pathology

**Figure 3:**
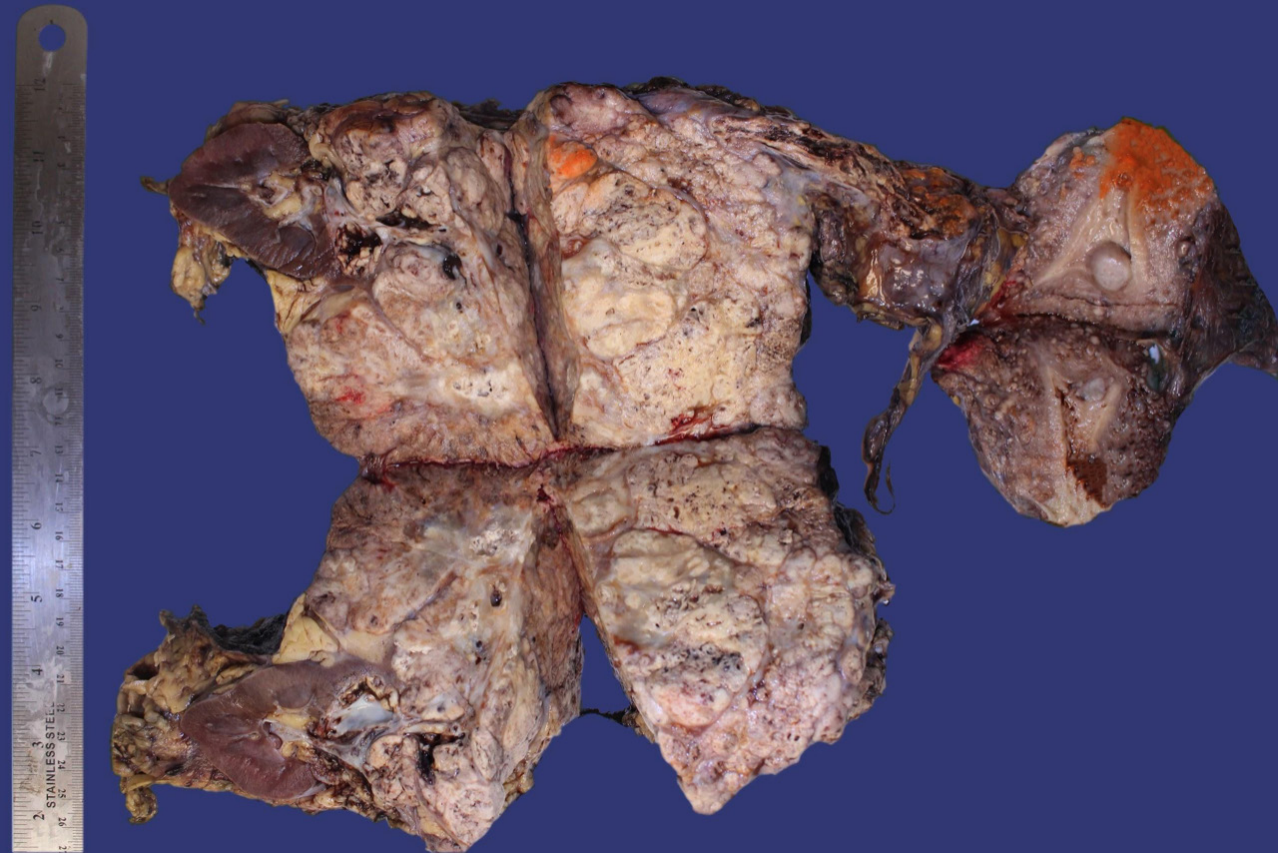
Gross examination of the surgical specimen revealed a large necrotic left renal mass extending via the gonadal vein to the uterus and bilateral ovaries.

The left kidney, supracervical uterus, bilateral fallopian tubes, and ovaries were sent for histopathological examination. The left kidney measuring 21.0 cm in greatest dimension showed a 15.6 × 11.2 × 11.1 cm yellow, gelatinous, hemorrhagic, and necrotic mass that obliterated the lower pole. The mass invaded the perinephric fat abutting Gerota’s fascia, the hilar fat, calyces, and distorted the renal sinus (Figure 3). The proximal renal vein contained tumor, which obliterated its lumen up to the point of branching with the left ovarian vein, and then tumor thrombus obliterated the left ovarian vein while the stump of the ovarian vein was patent. Grossly, the uterus showed approximately 70% of the endomyometrium involved by the tumor and necrosis, with diffuse tan-white nodularity on the serosal surface (Figure 3). The endometrium showed a striated and irregular appearance. Sectioning of the left fallopian tube showed edema, a pinpoint lumen, and tumor invasion. The left ovary showed invasion of the mass, with the remaining ovarian parenchyma estimated at 20%. The right fallopian tube was filled with a sanguinous fluid without clear evidence of gross tumor involvement, but the right ovary showed invasion by the mass. The uterus was also noted to contain leiomyoma and a microscopic focus of complex hyperplasia with atypia. Congo red stain was negative for amyloid.

**Figure 4:**
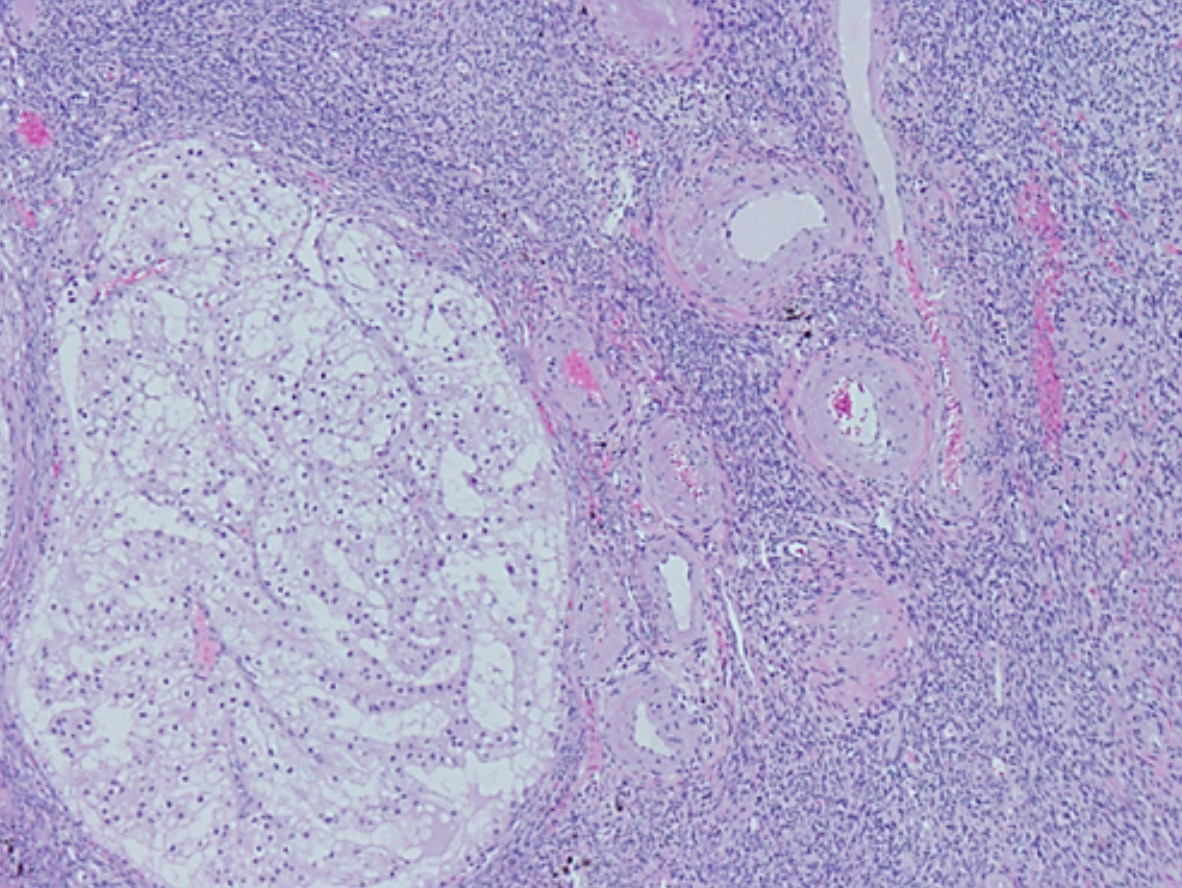
Microscopic evaluation of the tumor revealed nests of clear cells surrounded by interconnecting vascular framework, consistent with the ultimate diagnosis of clear cell renal cell carcinoma.

Microscopic examination revealed a tumor composed of nests of clear cells surrounded by interconnecting vascular framework. Cells with eosinophilic cytoplasm were also noted in combination with the clear cells. Immunohistochemical stains, including vimentin, were positive, and CD10 showed diffuse membranous positivity. Eosinophilic nucleoli were identifiable at 100×, consistent with Grade 3 tumor using the International Society of Urological Pathology (ISUP)/WHO grading scheme (13). No sarcomatoid or rhabdoid areas were noted. These findings rendered a diagnosis of left kidney clear cell RCC, which extended into the renal vein, left ovarian vein and uterus, left fallopian tube, and bilateral ovaries (Figure 4). The final pathologic stage was pT4N1M1.

## Discussion

To our knowledge, there have been no previously reported cases of RCC with direct contiguous invasion into the ipsilateral fallopian tube, uterus, and bilateral ovaries occurring along the pathway of the gonadal vein. The mechanism for the involvement of the contralateral ovary in this case is unclear, as there was no evidence of invasion into the associated fallopian tube. There may have been an extension from the uterus to the ovary, or it may have been a result of hematogenous metastasis similar to the nodal disease noted at initial imaging and the distant pulmonary metastasis, which were ultimately discovered postoperatively. Metastasis to the ovaries or fallopian tubes, although rare, has been described and appears to be reported more commonly than direct extension of the tumor (11, 12). The renal-ovarian axis has been proposed as the mechanism for metastasis given that the left gonadal vein drains into the left inferior vena cava; however, this theory would not explain metastasis to the right ovary (14).

## Conclusion

We presented a case of local extension of RCC through the gonadal vein into the ipsilateral ovary, fallopian tube, and uterus, which has not been previously described. Based on the available histopathologic data, we suspect that the uterus, left ovary, and left fallopian tube were involved by contiguous spread, while the right ovary, lymph nodes, and pulmonary metastases were likely a result of hematogenous spread. This tumor was managed with surgical resection followed by adjuvant immunotherapy. At the most recent follow-up, the patient was doing well without evidence of new metastatic disease.
